# Early presentations of dementia across cultures: Analysis of primary care data from a diverse population

**DOI:** 10.1002/alz.086108

**Published:** 2025-01-03

**Authors:** Sedigheh Zabihi, Jonathan P Bestwick, Phazha LK Bothongo, Qiqi Zhang, Christine Carter, Moïse Roche, Sarah Morgan‐Trimmer, Yvonne Birks, Mark Wilberforce, Ruth Dobson, Alastair J Noyce, John Robson, Fiona M Walter, Claudia Cooper, Charles R Marshall

**Affiliations:** ^1^ Queen Mary University of London, London United Kingdom; ^2^ University of Exeter, Exeter United Kingdom; ^3^ University of York, York United Kingdom; ^4^ Queen Mary University of London, London, England United Kingdom

## Abstract

**Background:**

Dementia is associated with a range of non‐cognitive features that can occur during the prodromal phase. Improved recognition of non‐cognitive presentations of dementia could reduce inequalities in dementia diagnosis, particularly if sociocultural factors influence rates of help‐seeking for cognitive symptoms. We aimed to investigate presentations to primary care in the years before dementia diagnosis in a deprived and ethnically diverse population with universal access to health care.

**Method:**

We conducted a nested case‐control study using electronic health care records on 1,016,277 individuals from primary care practices in East London, UK between 1990 and 2018. Individuals with a diagnosis of dementia were compared to controls without dementia. We ran a matched analysis (four controls matched to each person with dementia according to age and sex) using multivariable logistic regression to assess the associations between pre‐diagnostic presentations to primary care with subsequent diagnosis of dementia. We analysed three time periods (<2, 2‐5, 5‐10) before diagnosis.

**Result:**

We included 4,137 individuals and 15,754 controls in the matched analysis (51.8% White; 42.7% Black, South Asian and other ethnicities). Neuropsychiatric presentations including depression (odds ratio [OR] = 2.71; 95% CI: 2.33 to 3.16), anxiety (OR = 1.67; 95% CI: 1.46 to 1.92), initiation of antipsychotic medication (as a proxy for psychotic symptoms) (OR = 5.84; 95% CI: 4.80 to 7.11) and insomnia (OR = 1.69; 95% CI: 1.31 to 2.17) were more frequent in the years before dementia diagnosis. Associations were found for autonomic features including constipation (OR = 1.54; 95% CI: 1.39 to 1.71) and incontinence (OR = 2.63; 95% CI: 2.32 to 2.97) up to a decade, and hypotension (OR = 1.89; 95% CI: 1.35 to 2.63) up to five years before dementia diagnosis. Sensory features including hearing loss (OR = 1.36; 95% CI: 1.14 to 1.63), imbalance and dizziness were also associated with subsequent dementia diagnosis. Memory difficulty was more strongly associated with subsequent dementia diagnosis than any non‐cognitive presentation (OR = 51.00; 95% CI: 43.87 to 59.29).

**Conclusion:**

A range of non‐cognitive presentations are seen during the prodromal period of dementia in a diverse population. Improved recognition of these associations could increase access to dementia diagnosis, through improved recognition of potential presenting symptoms in people from different backgrounds.

